# Leatherback Turtle Movements, Dive Behavior, and Habitat Characteristics in Ecoregions of the Northwest Atlantic Ocean

**DOI:** 10.1371/journal.pone.0091726

**Published:** 2014-03-19

**Authors:** Kara L. Dodge, Benjamin Galuardi, Timothy J. Miller, Molly E. Lutcavage

**Affiliations:** 1 Department of Biological Sciences, University of New Hampshire, Durham, New Hampshire, United States of America; 2 Large Pelagics Research Center, University of Massachusetts Amherst, Gloucester, Massachusetts, United States of America; 3 National Marine Fisheries Service, Northeast Fisheries Science Center, Woods Hole, Massachusetts, United States of America; Institut Pluridisciplinaire Hubert Curien, France

## Abstract

Leatherback sea turtles, *Dermochelys coriacea*, are highly migratory predators that feed exclusively on gelatinous zooplankton, thus playing a unique role in coastal and pelagic food webs. From 2007 to 2010, we used satellite telemetry to monitor the movements and dive behavior of nine adult and eleven subadult leatherbacks captured on the Northeast USA shelf and tracked throughout the Northwest Atlantic. Leatherback movements and environmental associations varied by oceanographic region, with slow, sinuous, area-restricted search behavior and shorter, shallower dives occurring in cool (median sea surface temperature: 18.4°C), productive (median chlorophyll *a*: 0.80 mg m^−3^), shallow (median bathymetry: 57 m) shelf habitat with strong sea surface temperature gradients (median SST gradient: 0.23°C km^−1^) at temperate latitudes. Leatherbacks were highly aggregated in temperate shelf and slope waters during summer, early fall, and late spring and more widely dispersed in subtropical and tropical oceanic and neritic habitat during late fall, winter and early spring. We investigated the relationship of ecoregion, satellite-derived surface chlorophyll, satellite-derived sea surface temperature, SST gradient, chlorophyll gradient and bathymetry with leatherback search behavior using generalized linear mixed-effects models. The most well supported model showed that differences in leatherback search behavior were best explained by ecoregion and regional differences in bathymetry and SST. Within the Northwest Atlantic Shelves region, leatherbacks increased path sinuosity (i.e., looping movements) with increasing SST, but this relationship reversed within the Gulf Stream region. Leatherbacks increased path sinuosity with decreasing water depth in temperate and tropical shelf habitats. This relationship is consistent with increasing epipelagic gelatinous zooplankton biomass with decreasing water depth, and bathymetry may be a key feature in identifying leatherback foraging habitat in neritic regions. High-use habitat for leatherbacks in our study occurred in coastal waters of the North American eastern seaboard and eastern Caribbean, putting turtles at heightened risk from land- and ocean-based human activity.

## Introduction

Highly migratory marine predators such as leatherback sea turtles encounter a diversity of habitats during their long-distance movements. Oceanographic processes create regional ecosystems with distinct rates of primary productivity and community structure [1]. Predators may exhibit different behaviors in response to region-specific environmental conditions, with some regions optimal for foraging and (or) breeding while others serve as migratory habitat between breeding and feeding grounds. Obtaining direct measurements of foraging behavior in migratory marine species is challenging since the animals are difficult to observe for extended periods of time. Studies often rely on measures of search behavior to distinguish foraging from transiting, with the underlying assumption that a foraging animal should increase time and search effort in resource-rich areas (i.e., area-restricted search behavior) and decrease search effort in areas with fewer resources [2]. Marine animal tracking data has been used to measure area-restricted search behavior through analyses of speed, turning angle, path straightness and first passage time [3]–[5], while switching state-space models have been used to statistically estimate animal behavioral modes (e.g., foraging vs. transiting) [6]–[8].

Leatherback sea turtles (*Dermochelys coriacea*) are far-ranging marine predators, capable of swimming thousands of kilometers between boreal and tropical latitudes [9]–[11]. In recent decades, satellite telemetry has demonstrated that leatherbacks can undertake annual migrations (defined here as the seasonal movement between regions/habitats based on favorable versus unfavorable conditions, after Dingle & Drake [12]) in the Atlantic, Pacific and Indian Oceans [13]–[19]. These extensive migrations take leatherbacks through a heterogeneous seascape where they experience strong differences in biological and physical oceanographic conditions. In oceanic habitat, inferred foraging behavior and foraging success of some leatherbacks have been linked to fronts, upwelling and downwelling zones, and mesoscale features [15], [19]–[26], while some individuals move continuously without associating with particular oceanographic features [27]. Although leatherbacks are most often associated with an oceanic lifestyle, some individuals make seasonal use of highly productive continental shelf and slope habitats [16], [19], [26], [28]–[31], residing in near-shore areas for several months [18], [32]–[33]. Continental shelf and slope waters are productive regions where spring bloom conditions can lead to increased seasonal abundance of plankton [29], [34]–[36]. Increased nutrient input from land, tidal-mixing, and wind-driven upwelling can trigger increases in scyphozoan populations, while physical discontinuities in shelf waters and along ocean fronts promote aggregation and retention of gelatinous organisms [37]–[41]. Productive water masses and fronts in oceanic and neritic regions are also targeted by commercial fishing operations with fixed and mobile gear, often resulting in incidental captures of sea turtles and other non-target species [42]–[46].

Broad-scale tracking studies over the past decade have given new insight into the relationship between leatherback behavior and their environment [8], [17], [19], [21], [27], [47]–[52], but most of these studies focused on the post-nesting migrations of adult females. Comparatively few studies have been initiated in leatherback foraging grounds where different sexes and age classes mix [18]–[19], [24], [47]. We need additional data on males and females at multiple life history stages to fully understand the habitat-use and environmental associations of leatherback in-water populations.

In the present study, we deployed satellite tags on adult male, female and sub-adult leatherbacks turtles captured off Massachusetts, USA. This is the first in-water tagging study conducted in leatherback foraging grounds in the US Atlantic, and one of three direct-capture studies of leatherback turtles worldwide. We collected geolocation and dive data to: 1) determine leatherback occupancy of distinct oceanographic regions in the North Atlantic; 2) characterize leatherback regional movements, dive behavior, and environmental associations; 3) identify seasonal high-use habitat and 4) determine key environmental features associated with leatherback search behavior in the NW Atlantic.

## Methods

### Ethics statement

This work was conducted under the authority of the National Marine Fisheries Service Endangered Species Act Section 10 Permit # 1557-03 and the University of New Hampshire IACUC # 060501 and #090402. Turtle disentanglement was conducted under the authority of NOAA 50 CFR Part 222.310.

### Satellite telemetry

**Figure 1:**
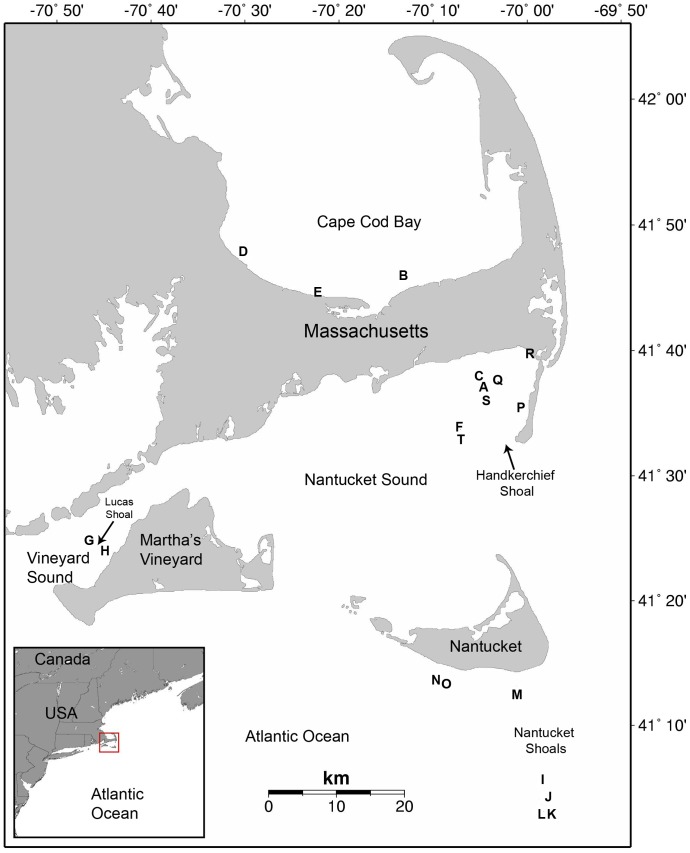
Leatherback turtle tagging locations off Cape Cod, Massachusetts, USA, 2007–2009. Letters represent satellite tag deployments as listed in Table 1.

Leatherbacks were located off the coast of Massachusetts, USA (∼41°N, 70°W) from August 2007 to September 2009, and captured with a breakaway hoopnet (n = 11) [53] or accessed through the Massachusetts sea turtle disentanglement network (n = 9) (Fig. 1; Table 1). The breakaway hoopnet was deployed from a modified bowsprit on commercial fishing or research vessels, and the net was pursed over individual turtles at the surface. We moved netted turtles alongside the vessel to the stern and brought them on board using a custom-built stern ramp. Veterinary personnel conducted health evaluations on all turtles prior to tag attachment [54].

**Table 1:** Summary data for twenty leatherback sea turtles equipped with satellite tags off Massachusetts, USA, 2007–2009.

Turtle ID	PTT^1^ Number	CCL^2^ (cm)	Age^3^	Sex^4^	Capture method	Tag Model^5^	Tagging location^6^	Tagging date	Days at liberty	Distance (km)	No. ARGOS locations	No. GPS locations
A	68366	140.7	S	M	Entangled	MK10-AF	N. Sound	19-Aug-07	34	938	137	263
B	68364	143.2	S	M	Entangled	MK10-AF	CC Bay	29-Aug-07	18	461	82	90
C	68369	123.0	S	U	Entangled	MK10-AF	N. Sound	29-Aug-07	16	277	64	89
D	68370	137.5	S	U	Entangled	MK10-AF	CC Bay	22-Sep-07	183	6444	572	777
E	68365	136.0	S	F	Entangled	MK10-AF	CC Bay	1-Oct-07	35	991	109	252
F	68365a	149.5	A	M	Hoopnet	MK10-AF	N. Sound	17-Jul-08	174	8004	1520	1067
G	68364a	146.0	A	F	Hoopnet	MK10-AF	V. Sound	26-Jul-08	199	7920	1407	1498
H	82052	161.5	A	F	Hoopnet	MK10-A	V. Sound	29-Jul-08	272	8435	2114	na
I	76988	152.2	A	M	Hoopnet	MK10-AF	Nantucket	10-Aug-08	214	8878	1932	1469
J	76990	140.4	S	U	Hoopnet	MK10-AF	Nantucket	10-Aug-08	150	5967	1147	593
K	82055	133.8	S	U	Hoopnet	MK10-A	Nantucket	10-Aug-08	152	5792	1306	na
L	82051	153.3	A	M	Hoopnet	MK10-A	Nantucket	10-Aug-08	242	9466	1846	na
M	76989	144.8	A	F	Hoopnet	MK10-AF	Nantucket	21-Aug-08	180	9191	1427	1563
N	85538	154.0	A	M	Hoopnet	MK10-AF	Nantucket	22-Aug-08	183	6528	1707	796
O	85537	138.5	S	M	Hoopnet	MK10-AF	Nantucket	22-Aug-08	181	5883	1722	307
P	82053	146.4	A	M	Entangled	MK10-A	N. Sound	23-Aug-08	234	9765	1095	na
Q	82054	140.0	S	U	Entangled	MK10-A	N. Sound	28-Aug-08	191	5980	1306	na
R	82056	126.5	S	U	Weir	MK10-A	N. Sound	10-Jul-09	414	14168	3570	na
S	82057	127.7	S	U	Hoopnet	MK10-A	N. Sound	27-Aug-09	278	11541	1616	na
T	27579	155.0	A	M	Entangled	MK10-A	N. Sound	3-Sep-09	203	7096	1458	na

1 PTT: platform transmitter terminal.

2 CCL: curved carapace length.

3 S: sub-adult (<145 cm CCL); A: adult (≥145 cm CCL).

4 M: male; F: female, U: unknown sex.

5 MK10-A: Argos-only locations; MK10-AF: Argos and Fastloc GPS locations.

6 N. Sound: Nantucket Sound; CC Bay: Cape Cod Bay; V. Sound: Vineyard Sound; Nantucket: waters south of Nantucket.

Twenty leatherbacks were fitted with Wildlife Computers, Inc. (Redmond, WA, USA) model MK10-A (n = 8) and MK10-AF (n = 12) ARGOS-linked satellite time depth recorders. The tags deployed in 2007 had flexible, plastic-coated metal baseplates that conformed to the leatherbacks' medial ridge. We worked with Wildlife Computers to improve this design in future seasons (2008–2009), resulting in the “ridge-mount” tag model specifically developed for leatherback turtles. We attached satellite tags directly to the leatherbacks' medial ridge following methods first developed by Lutcavage et al. [55]. Prior to attachment we cleaned and anesthetized the attachment site with Betadine (three applications), isopropyl alcohol and a topical freezing agent (ethyl chloride) [55]. We made two horizontal drill holes (4.5 mm diameter) in the ridge bone using an electric drill and orthopedic drill bit. Drill hole sites were marked with furazone ointment and spaced to match the spacing of the MK10-A and MK10-AF ridge-mount tag bases. Plastic-coated braided stainless steel tethers were passed through tygon sheathing in the two drill tracts. We created a base for the tag that conformed to the turtle's ridge using a two-part cold-curing silicone putty (Equinox Silicone Putty, Smooth-On, Inc., Easton, PA, USA), and the tag was positioned on top of the putty base. The two tethers were threaded through holes in the tag base and secured using corrodible stainless steel crimps to ensure eventual release of the tag.

Leatherbacks were measured to the nearest 0.1 cm (curved carapace length: CCL and curved carapace width: CCW) with a flexible fiberglass measuring tape, and ranged from 123.0 to 161.5 cm CCL (Table 1). We used CCL to classify turtles as adults (CCL≥145 cm) or sub-adults (CCL<145 cm), and we determined gender based on tail length of adult turtles [56]. Five sub-adult turtles were sexed based on presence of a penis, subsequent necropsy or evidence of nesting. We collected samples of blood and skin [54], [57], and all turtles were photographed, scanned for passive integrated transponder (PIT) tags, and given PIT and flipper tags if none were present.

The satellite tags transmitted Fastloc GPS locations (MK10-AF model only), ARGOS-derived locations (all tags) and dive information (depth resolution ±0.5m and temperature resolution ±0.05°C) via Service ARGOS (Toulouse, France) (Table 1). Ninety-five percent of Fastloc GPS locations are accurate to ±55 m [58], while ARGOS-derived location error varies by location class (LC) as follows: LC3<150 m, LC2 150–350 m, LC1 350–1000 m, and LC0>1000 m. ARGOS does not provide accuracy estimates for LCA and LCB locations, and LCZ are considered invalid.

We defined a dive as vertical movement below two meters for at least one minute. The number of dives within specified depth and duration ranges and the time spent within depth and temperature ranges were collected as frequency histograms based on preprogrammed bins (Table 2). Histograms were aggregated over four 6-hour periods in GMT: 0:00–5:59, 6:00–11:59, 12:00–17:59, 18:00–23:59. Tags deployed in 2007 and 2008 (n = 17) were programmed to transmit daily while tags deployed in 2009 (n = 3) were programmed to transmit daily from July to December, every other day from January to April, and every third day from May to June.

**Table 2:** Bin ranges of dive parameters from satellite tags deployed on leatherback sea turtles, 2007–2009.

Years	Number of tags	Depth bin (m)	Duration bin (min)	Years	Number of tags	Time-at-Depth bin (m)	Time-at-Temp bin (°C)
2007–2009	20	2–5	1–4	2008–2009	15	0–2	0–4
2007–2009	20	5–10	4–8	2008–2009	15	2–10	4–6
2007–2009	20	10–15	8–12	2008–2009	15	10–15	6–8
2007–2009	20	15–20	12–16	2008–2009	15	15–20	8–10
2007–2009	20	20–25	16–20	2008–2009	15	20–25	10–12
2007–2009	20	25–30	20–24	2008–2009	15	25–30	12–14
2007–2009	20	30–50	24–28	2008–2009	15	30–40	14–16
2007–2009	20	50–75	28–32	2008–2009	15	40–50	16–18
2007–2009	20	75–100	32–36	2008–2009	15	50–75	18–20
2007–2009	20	100–200	36–40	2008–2009	15	75–100	20–22
2007–2009	20	200–300	40–44	2008–2009	15	100–125	22–24
2007–2009	20	300–400	44–48	2008–2009	15	125–150	24–26
2007–2009	20	400–500	48–52	2008–2009	15	150–200	26–28
2007–2009	20	>500	>52	2008–2009	15	>200	>28

### Environmental data

We selected environmental data likely to influence production and distribution of gelatinous prey [39], [59] and thus leatherback movements. Sea surface temperature (SST), surface chlorophyll *a* concentration (chl *a*), SST gradient, chl *a* gradient and bathymetry were used as potential predictors. We used SST and chl *a* gradients as a proxy for the presence of fronts [60]. SST data were obtained as a blended product available at the GHRSST website (http://www.ghrsst.org) as 9 km, daily averages. The blended SST product was derived from microwave SST data from three sources (Advanced Microwave Scanning Radiometer, Tropical Rainfall Measuring Mission Microwave Imager and WindSAT Polarimetric Radiometer) and InfraRed SST data from Aqua Moderate Resolution Imaging Spectroradiometer (MODIS). Chl *a* data were obtained from MODIS data as 2.5 km, 8 day averages at the NOAA ERDDAP website (http://www.coastwatch.pfeg.noaa.gov/erddap/info/erdMWchla8day/index.html). Bathymetry was determined using 1-minute gridded global relief data (ETOPO1) from the National Geophysical Data Center (http://www.ngdc.noaa.gov/mgg/global/). SST and chl *a* gradients were generated using the Belkin-O'Reilly oceanic front detection algorithm [60].

### Data analysis

We filtered 30,173 raw ARGOS and GPS locations using Kalman filter methods outlined in Royer & Lutcavage [61]. Since our analysis included GPS data, we extended the original error covariance structure to include this information. Data were interpolated to a three-hour time step and smoothed. Environmental data were then extracted for the 28,253 filtered turtle locations. Rate of travel was determined using the distance function in Matlab (Mathworks, Natick, MA) and a daily straightness index was calculated as the ratio of straight-line distance to total distance traveled by each turtle per day (in km d^−1^), resulting in a dimensionless index from near 0 (sinuous) to 1 (straight) [62]–[63]. Travel rate and straightness were not calculated from January to June for the three turtles tagged in 2009 since tags were duty-cycled and did not transmit daily during these months. Leatherback positions and dive data were assigned to distinct biogeographic provinces or “ecoregions” defined by Longhurst [1]. We calculated the duration of leatherback occupancy in each ecoregion, and assessed variability in leatherback search effort, dive behavior and environmental associations across ecoregions. Averages are shown as mean ± standard deviation (mean ± SD) for normally distributed data, and median, interquartile range (Q1–Q3) where data are not normally distributed.

To investigate variation in seasonal habitat use, we created density utilization maps of filtered leatherback positions for pooled data across all turtles by season. Seasons were defined as: July – September (summer), October – December (autumn), January – March (winter), and April – June (spring). Daily locations were summed into hexagonal area bins, with the area of each hexagon approximately 669 km^2^ (or 4 hexagons per degree). These bins are larger than the error associated with our filtered ARGOS and GPS location data, but small enough to identify regional high-use areas. Density utilization maps were produced using R [64] and Generic Mapping Tools [65] (see http://www.scott.sherrillmix.com/R-GMT_HexPlot.php).

We applied generalized linear mixed-effects models to investigate the influence of ecoregion, SST, chl *a*, SST gradient magnitude, chl *a* gradient magnitude and bathymetry (fixed effects) on leatherback path straightness while accounting for the correlation of repeated observations from individual turtles [66]. Changes in path straightness have been used to identify purported search behavior associated with foraging in leatherbacks [25], as well as other marine predators [67]–[69]. Density plots of the environmental data showed that logarithmic transformation was required for SST gradient, chl *a*, chl *a* gradient and bathymetry. We were primarily interested in the influence of SST, SST gradient, chl *a* and chl *a* gradient in regions where these surface features are most variable (least homogenous), and we were mainly interested in the effect of bathymetry in neritic habitats where leatherbacks can access the entire water column. Therefore, we estimated region-specific regression parameters for these variables: SST (Northwest Atlantic Shelves, Gulf Stream), SST gradient (Northwest Atlantic Shelves, Gulf Stream) and bathymetry (Northwest Atlantic Shelves, Guianas Coastal). We compared models where effects of SST, SST gradient, chl *a*, chl *a* gradient and bathymetry on path straightness are the same for the respective groups of regions with less parsimonious models that allow the effects to differ for each ecoregion (Table 3). As path straightness ranges from near 0 to 1, we assumed a Gaussian error structure for the logit-transformed path straightness with continuous first-order autoregressive model correlation structure for repeated observations as a function of time between observations [70].

**Table 3:** Definition of fitted mean logit path straightness models for observation *j* of turtle *i*.

Model	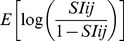
null	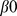
ecoregion	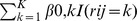
ecoregion + SST^a^	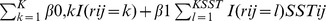
ecoregion × SST^b^	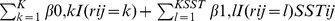
ecoregion + chla^a^	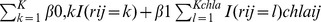
ecoregion × chla^b^	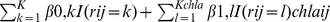
ecoregion + SSTg^b^	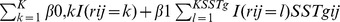
ecoregion × SSTg^b^	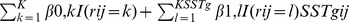
ecoregion + chlag^b^	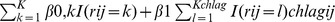
ecoregion × chlag^b^	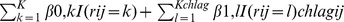
ecoregion + bathymetry^a^	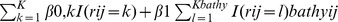
ecoregion × bathymetry^b^	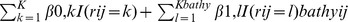
ecoregion + SST^a^ + bathymetry^a^	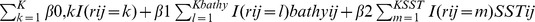
ecoregion × SST^b^ + bathymetry^a^*	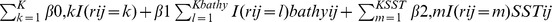
ecoregion + chla^a^ + bathymetry^a^	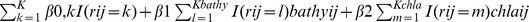
ecoregion × chla^b^ + bathymetry^a^	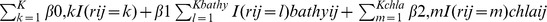
ecoregion + SSTg^a^ + bathymetry^a^	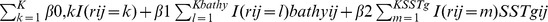
ecoregion × SSTg^b^ + bathymetry^a^	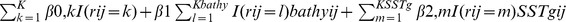
ecoregion + chlag^a^ + bathymetry^a^	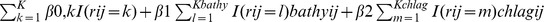
ecoregion × chlag^b^ + bathymetry^a^	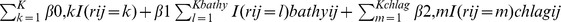

Turtle *i* is in ecoregion *r_ij_* at observation *j*, *I*(*x*) is an indicator function equaling 1 when x is true and 0 otherwise. There are *K* ecoregions, and *K_SST_*, *K_SSTg_*, *K_chla_*, *K_chlag_*, and *K_bathy_* are the number of ecoregions where SST, SSTg, chla, chlag, and bathy effects are allowed. The variance structure is the same for all models.

a Slopes held constant in regions of interest.

b Slopes and intercepts allowed to vary in regions of interest.

We fit the models by maximum marginal likelihood in R [64] using the lme4 package and compared relative performance using Akaike Information Criterion (AIC) [71] of the fitted models. As a measure of evidence for relative performance of each model that we fit, we used Akaike weights [72]. We chose to use AIC over a criteria adjusted for sample size (AIC_c_) because AIC_c_ requires a known number of independent observations, and this is not straightforward for mixed-effects models [73]. For first-order autoregressive models as used here, the effective number of independent observations depends on the correlation of the observations within each individual. Since the autocorrelation we estimated for our models is generally low, we had a large effective number of independent observations where differences between AIC and AIC_c_ are negligible. For the model that provided the best fit, we used restricted maximum likelihood to obtain parameter estimates and predict changes in steepness with various covariates and factors [74].

## Results

### Satellite telemetry

**Figure 2:**
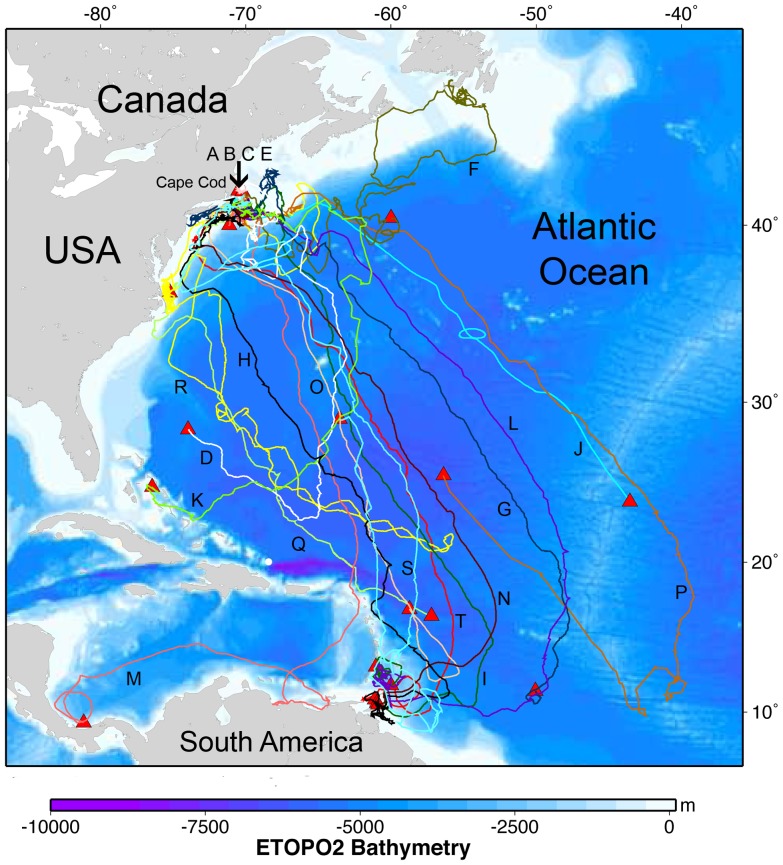
Reconstructed movements of 20 satellite-tagged leatherback turtles in the North Atlantic Ocean, 2007–2010. Tracks show turtle movements from point of release (Cape Cod) to point of last Argos transmission (red triangles). Tags were deployed on adult males (F, I, L, N, P, T), adult females (G, H, M), and sub-adults (A, B, C, D, E, J, K, O, Q, R, S).

**Figure 3:**
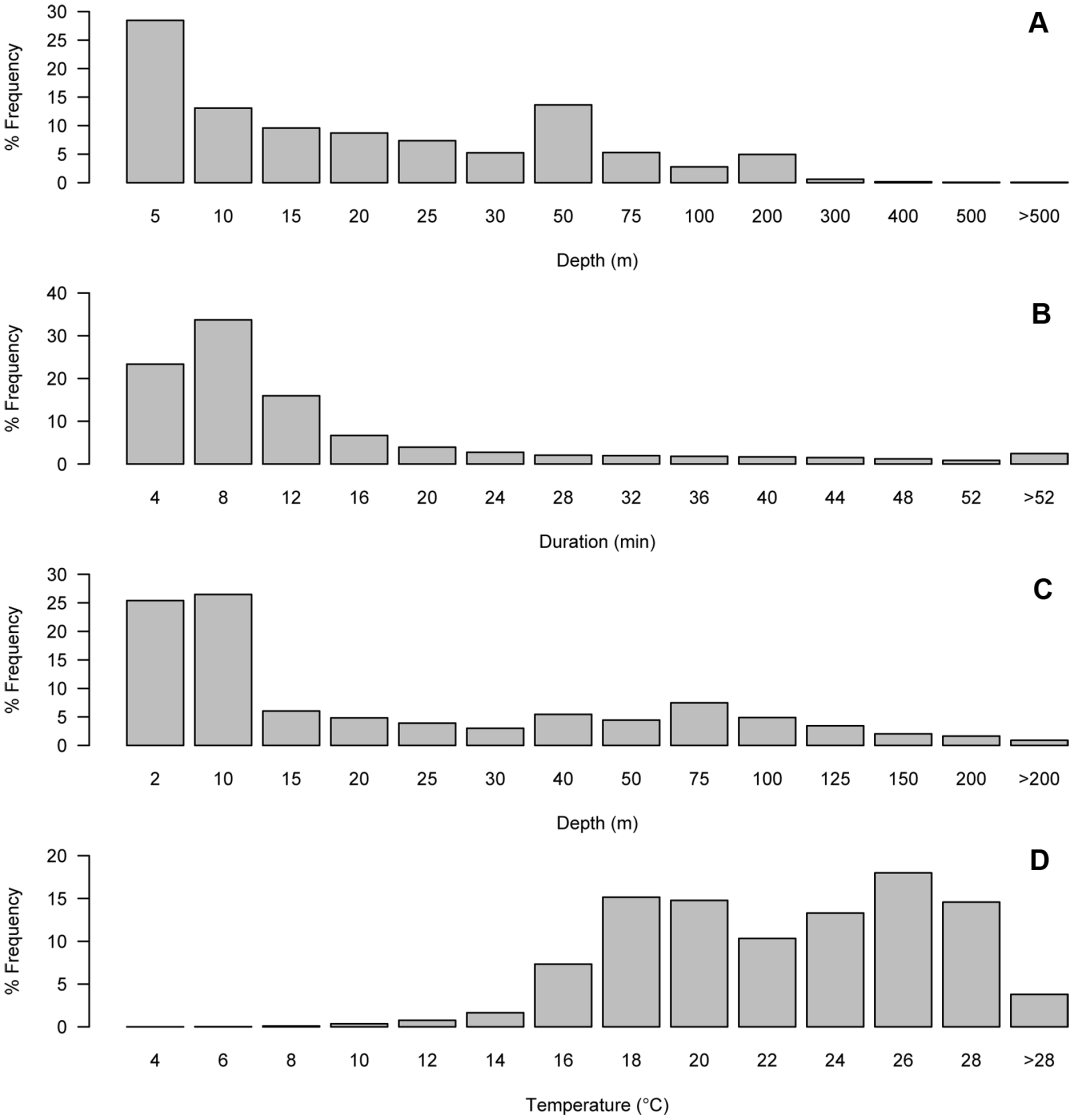
Frequency distributions of dive parameters from satellite tags on leatherback turtles in the North Atlantic Ocean. a) Dive-depth (n = 19) and b) dive-duration (n = 17) from turtles tagged 2007–2009. c) Time-at-depth (n = 15) and d) time-at-temperature (n = 15) from turtles tagged from 2008–2009.

We received data from all tagged leatherbacks: four tags transmitted for less time than expected and 16 tags met or exceeded predicted battery life. Tags reported between 16 and 414 days, with a median tracking duration of 184 (152 to 219; Q1–Q3) days (Fig. 2; Table 1). We pooled the percent frequency distributions of the four dive parameters: dive-depth (n = 19 tags), dive-duration (n = 17 tags), time-at-depth (n = 15 tags), and time-at-temperature (n = 15 tags), shown in Fig. 3. Three tags deployed in 2007 reported spurious dive-duration data (i.e., the total number of dives recorded in the >52 min bin exceeded the 6-hour time period) and one 2007 tag recorded insufficient dive-depth data, so these tags were excluded from the dive-duration and dive-depth analysis. We only included time-at-depth and time-at-temperature data for tags with the same lower bin ranges (2008 and 2009).

Of the 210,556 dives reported for the dive-depth parameter, over 28% were to depths less than 5 m and 90% were shallower than 75 m (Fig. 3a). Fifteen turtles dove deeper than 500 m during the study period, with males recording the deepest dives (>1200 m, n = 3 turtles). The pooled dive-duration data showed that close to 75% of all leatherback dives were less than 12 min, and more than 90% were shorter than 32 min (Fig. 3b). Sixteen turtles recorded extended dives lasting over 52 minutes, but these represent less than 3% of the total. The pooled frequency distribution of the time-at-depth shows that turtles spent over 25% of their time within 2 m of the surface, and over 50% of their time shallower than 10 m (Fig. 3c). Over 90% of their time was spent in the top 100 m of the water column. The pooled frequency distribution of the time-at-temperature shows that turtles spent 86% of their time between 16°C and 28°C (Fig. 3d).

### Habitat use and environmental associations

**Figure 4:**
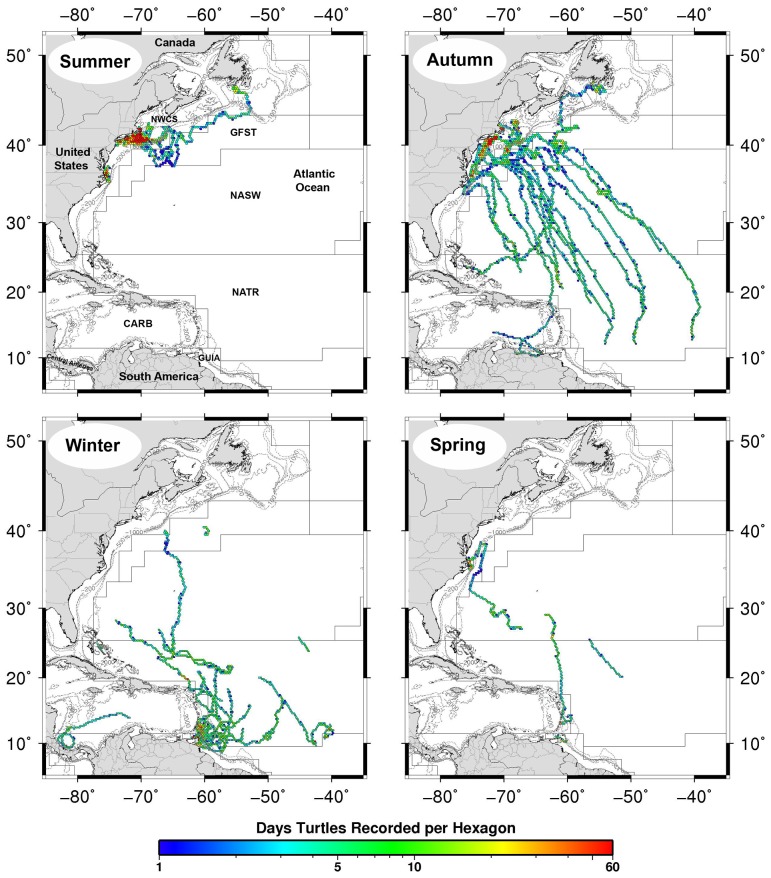
Seasonal habitat use of adult male, female and sub-adult leatherback turtles, 2007–2010. Summer, July – September (n = 19 turtles), autumn, October – December (n = 17 turtles), winter, January – March (n = 16 turtles), and spring, April – June (n = 5 turtles). There are four hexagons per degree; each hexagon represents approximately 669 km^2^. Color scale shows the number of track days per hexagon. Ecoregions from Longhurst [1]: NWCS, Northwest Atlantic Shelves; GFST, Gulf Stream; NASW, North Atlantic Subtropical Gyral West; NATR, North Atlantic Tropical Gyral; CARB, Caribbean; GUIA, Guianas Coastal.

Leatherbacks ranged widely between 39°W and 83°W, and between 9°N and 47°N (Fig. 2), over six oceanographically distinct ecoregions defined by Longhurst [1]: the Northwest Atlantic Shelves (n = 20), the Gulf Stream (n = 16), the North Atlantic Subtropical Gyral West (hereafter referred to as the Subtropical Atlantic, n = 15), the North Atlantic Tropical Gyral (hereafter referred to as the Tropical Atlantic, n = 15), the Caribbean (n = 6) and the Guianas Coastal (n = 7) (Fig. 4). All leatherbacks were tagged in the Northwest Atlantic Shelves, and 16 turtles (Turtles D, F–T) were tracked long enough to determine an average minimum residency of 79 days (±39 days) in this region post-tagging (Table 1). Fifteen turtles (Turtles D, G–T) left the Northwest Atlantic Shelves between late September and mid-November, with the majority leaving between mid-October and mid-November (n = 11, Turtles D, G–K, N–O, R–T). Most turtles spent less than a week in the Gulf Stream region (median 6 days), but two individuals (Turtles F & D; Table 1 & Fig. 2) made more extensive use of this region (95 and 59 days, respectively). Between October and February, leatherbacks transited rapidly through the Subtropical Atlantic (median 29 days), entering the Tropical Atlantic between November and early February. Leatherbacks either remained in the Tropical Atlantic for the remainder of the tracking period (n = 7, Turtles D, G, J–K, O–P, R) or continued on to breeding and (or) foraging areas in Caribbean and Guianas Coastal regions (n = 8, Turtles H–I, L–N, Q, S–T). Three sub-adults and one small adult male were tracked long enough to observe a complete (32 and 92 days; Turtles D & R, Table 1) or partial (83 and 56 days; Turtles P & S, Table 1) overwintering period in the Tropical Atlantic before they returned to the Subtropical Atlantic between late March and mid-May. One turtle (Turtle R) returned to the Northwest Atlantic Shelves in mid-May, remaining in the region for 96 days before the tag stopped transmitting in late August.

**Figure 5:**
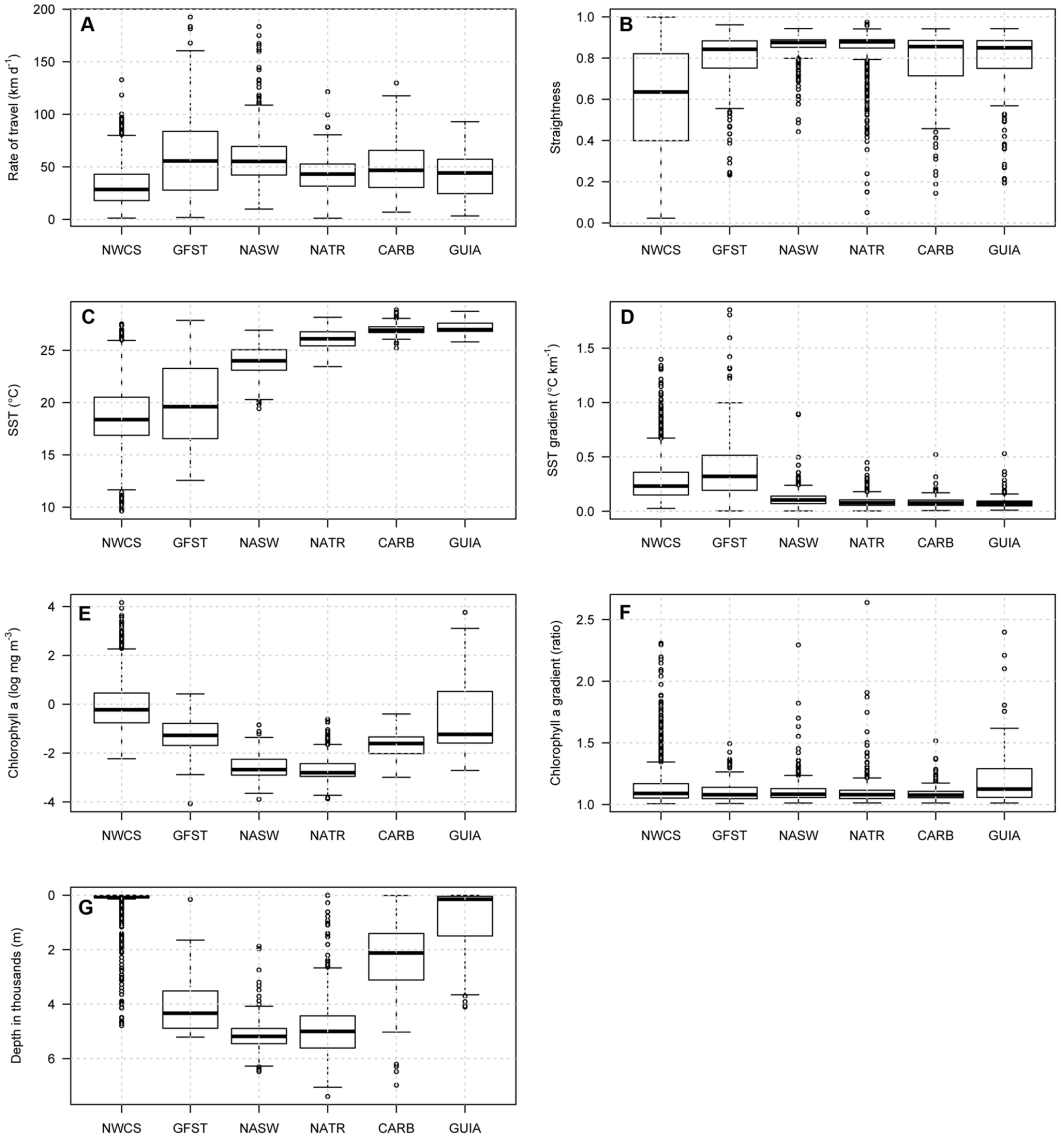
Leatherback behavioral indices in Longhurst regions of the North Atlantic Ocean. a) Rate of travel, b) straightness, c) SST, d) SST gradient magnitude, e) chl *a*, f) chl *a* gradient magnitude, and g) bathymetric depth. NWCS, Northwest Atlantic Shelves; GFST, Gulf Stream; NASW, North Atlantic Subtropical Gyral West; NATR, North Atlantic Tropical Gyral; CARB, Caribbean; GUIA, Guianas Coastal. Boxplots: centerline, median; edges of box, 1st and 3rd quartiles; whiskers, data points within the range Q1−1.5(Q3−Q1) to Q3+1.5(Q3−Q1).

**Figure 6:**
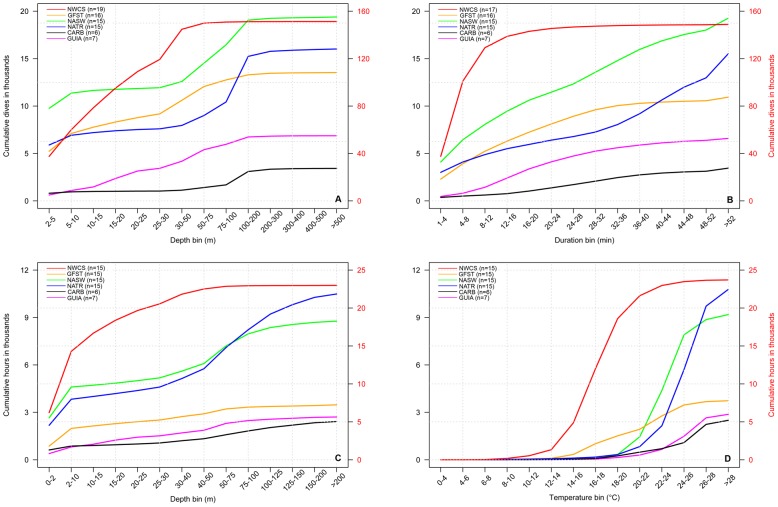
Cumulative frequency plots of leatherback dives in Longhurst regions of the North Atlantic Ocean. Depth and duration bins (top panels) and leatherback hours in depth and temperature bins (bottom panels). NWCS, Northwest Atlantic Shelves; GFST, Gulf Stream; NASW, North Atlantic Subtropical Gyral West; NATR, North Atlantic Tropical Gyral; CARB, Caribbean; GUIA, Guianas Coastal. The second y-axis (red) corresponds to NWCS (red line) while all other regions reference the first y-axis (black). This highlights the increased dive activity of tagged turtles in the NWCS region.

Turtles modified their movements and dive behavior while occupying different ecoregions. Leatherbacks in the Northwest Atlantic Shelves had the lowest travel rates and path straightness of all regions (Fig. 5a,b), and they combined slow, sinuous swimming with short, shallow dives (Fig. 6a,b). They spent most of their time in the top 10 m of the water column at temperatures between 16°C and 20°C (Fig. 6c,d). Outside of the Northwest Atlantic Shelves, turtles increased their travel rate and path straightness (Fig. 5a,b). Leatherbacks continued making shallow dives (<5 m), but increasingly made deeper, longer dives as they traveled south (Fig. 6a,b). As turtles moved into subtropical and tropical ecoregions, they began spending more time at temperatures over 22°C and at depths over 50 m, experiencing the warmest temperatures and making the deepest, longest dives in the Tropical Atlantic and Caribbean (Fig. 6c,d). Diving patterns in the primarily shelf waters of the Guianas Coastal region were distinct from other tropical regions: leatherbacks spread their diving effort throughout the top 200 m and most dives were less than 24 min (Fig. 6a,b). Leatherback surface times (0–2 m) were similar across most regions, with the greatest surface times recorded in the Subtropical Atlantic (mean 30%), Northwest Atlantic Shelves (mean 27%), Caribbean (mean 26%) and Gulf Stream (mean 25%). Leatherbacks spent the least amount of time at the surface in the Guianas Coastal (mean 14%) and Tropical Atlantic (mean 21%).

Turtles experienced highly variable environmental conditions across ecoregions, where bathymetry ranged from shallow bays and sounds on the continental shelf to deep oceanic waters, SST from 9.6°C to 28.9°C and chl *a* from near zero to 64.36 mg m^−3^ (Fig. 5). Leatherbacks occupied areas with SST and chl *a* gradients of varying magnitudes, with SST gradients of 0 to 1.85°C km^−1^ and chl *a* gradients of 1.052 to 1.144 (ratio) (Fig. 5). Turtles in the Northwest Atlantic Shelves used relatively shallow habitat, staying mostly within the 80 m isobath and associating with the highest chl *a* of all regions (Fig. 5e,g). Turtles experienced the coolest, most variable sea surface temperatures and strongest SST gradients in the Northwest Atlantic Shelves and the Gulf Stream (Fig. 5c,d). While the chl *a* gradients were similar across ecoregions, leatherbacks in the Guianas Coastal region used habitat with the most variable and strongest chl *a* gradients (Fig 5f).

**Figure 7:**
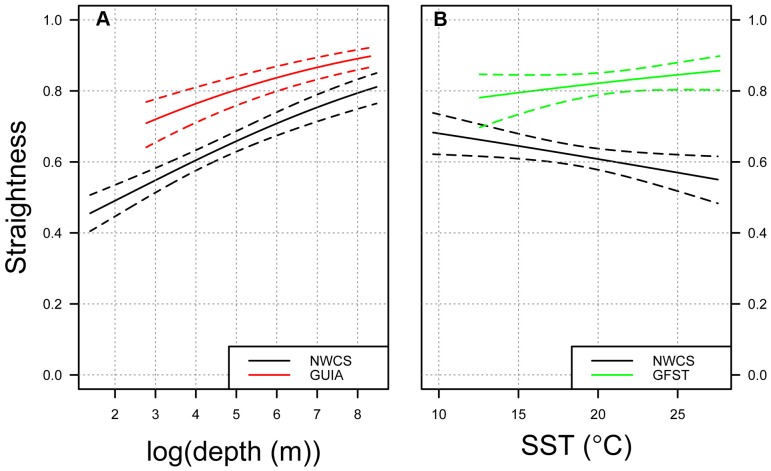
Predicted straightness (solid lines) and 95% confidence intervals (dashed lines) from the best performing model in Table 4. Leatherback path straightness is shown in relation to: a) observed log-transformed bathymetry (m) in each of two distinct ecoregions of the northwest Atlantic and b) observed sea surface temperature (SST) in each of two distinct ecoregions of the northwest Atlantic; Northwest Atlantic Shelves (NWCS); Gulf Stream (GFST); Guianas Coastal (GUIA). Mean SST value in each region was used to create the bathymetry plot and mean bathymetry value in each region was used to create the SST plot.

Based on AIC values, the most well supported model showed that differences in leatherback search behavior (represented by logit-transformed path straightness) were best explained by ecoregion and effects of bathymetry and SST, with effects of SST depending on the ecoregion (Table 4). In the mainly neritic ecoregions (Northwest Atlantic Shelves and Guianas Coastal), there was a positive relationship between path straightness and bathymetry, with leatherback movements becoming more sinuous as water depth decreased (Table 5; Fig. 7a). In the regions where SST was most variable (Northwest Atlantic Shelves and Gulf Stream), the relationship between path straightness and SST differed. In the Gulf Stream, the relationship between path straightness and SST was positive, with leatherback path sinuosity increasing with decreasing SST. In the Northwest Atlantic Shelves, the relationship between path straightness and SST was negative, with leatherbacks increasing path sinuosity with increasing SST (Table 5; Fig. 7b). The relationship between path straightness and SST in the Gulf Stream region was slightly positive, reflecting the fact that this slope parameter was not significant in the model.

**Table 4:** AIC results for linear mixed-effects models where mean logit-transformed path straightness is a function of ecoregion, sea surface temperature (SST), logarithm of surface chlorophyll a concentration (chla), logarithm of SST gradient (SSTg), logarithm of surface chla gradient (chlag), and logarithm of bathymetry (bathy).

Model	p^1^	AIC	ΔAIC^2^	ω^3^
null	4	7258.06	390.51	0
ecoregion	9	6927.63	60.08	0
ecoregion + SST^a^	10	6927.26	59.71	0
ecoregion × SST^b^	11	6921.62	54.07	0
ecoregion + chla^1^	10	6908.31	40.76	0
ecoregion × chla^b^	15	6908.97	41.42	0
ecoregion + SSTg^a^	10	6928.73	61.18	0
ecoregion × SSTg^b^	11	6929.90	62.35	0
ecoregion + chlag^a^	10	6923.94	56.39	0
ecoregion × chlag^b^	15	6922.50	54.95	0
ecoregion + bathymetry^a^	10	6871.17	3.62	0.07
ecoregion × bathymetry^b^	11	6873.15	5.60	0.03
ecoregion + SST^a^ + bathymetry^a^	11	6871.52	3.97	0.06
ecoregion × SST^b^ + bathymetry^a^ *	12	6867.55	0.00	0.42
ecoregion + chla^a^ + bathymetry^a^	11	6871.76	4.21	0.05
ecoregion × chla^b^ + bathymetry^a^	16	6868.89	1.34	0.21
ecoregion + SSTg^a^ + bathymetry^a^	11	6873.13	5.58	0.03
ecoregion × SSTg^b^ + bathymetry^a^	12	6874.93	7.38	0.01
ecoregion + chlag^a^ + bathymetry^a^	11	6871.61	4.06	0.05
ecoregion × chlag^b^ + bathymetry^a^	16	6870.84	3.29	0.08

1 p: number of parameters in the model.

2 ΔAIC: difference in AIC value between best fitting model and other model.

3 ω: Akaike weight.

a Slopes held constant in regions of interest.

b Slopes and intercepts allowed to vary in regions of interest.

*Best fitting model.

**Table 5:** Fixed effects parameter estimates of final model.

Effect	Estimate	Standard Error
Intercept	1.563	0.119
GFST^1^	−0.723	0.522
GUIA^2^	−1.312	0.235
NASW^3^	0.303	0.125
NATR^4^	0.337	0.121
NWCS^5^	−1.467	0.321
log(bathy)^6^	0.231	0.030
sst_NWCS^7^	−0.032	0.014
sst_GFST^8^	0.034	0.024

Random effect parameter estimate intercept was 0.196 and residual was 0.960, and estimated autocorrelation was 0.306.

1 GFST: Gulf Stream ecoregion.

2 GUIA: Guianas coastal ecoregion.

3 NASW: North Atlantic Subtropical Gyral West ecoregion.

4 NATR: North Atlantic Tropical Gryral ecoregion.

5 NWCS: Northwest Atlantic Shelves ecoregion.

6 log(bathy): logarithm of bathymetry in NWCS and GUIA ecoregions.

7 sst_NWCS: sea surface temperature in NWCS ecoregion.

8 sst_GFST: sea surface temperature in GFST ecoregion.

Seasonal density utilization maps showed leatherback movements were the least extensive during summer, with turtles tagged off Massachusetts showing a strong preference for the Northeast US continental shelf, concentrating movements off southern New England and Virginia/North Carolina (Fig. 4 ‘Summer’). Leatherbacks expanded their range in autumn, increasing their use of the Mid-Atlantic Bight and Gulf Stream before initiating a rapid, directed southward migration, following widely dispersed pathways through the Subtropical and Tropical Atlantic (Fig. 4 ‘Autumn’). Winter movements were restricted to tropical oceanic habitat and neritic waters of the Antilles, South America, and Central America, with the exception of two sub-adults (Turtles D & R) and one small adult male (Turtle F) that occupied subtropical oceanic waters for part of the season. Densely occupied winter habitat occurred off breeding beaches in the Windward Islands, particularly the north/northeast coast of Trinidad and the western half of the Tobago Basin (Fig. 4 ‘Winter’). Our limited tracking data for the spring showed that leatherbacks either remained in tropical breeding areas in the Windward Islands or began northward migrations, with one individual (Turtle R) occupying the North Carolina shelf (Fig. 4 ‘Spring’).

Overall, dispersal patterns differed between adult and sub-adult leatherbacks. Most adults followed widely spaced but highly oriented south/southeast headings during their southward migration until they reached latitudes between 10°N and 13°N (Fig. 2). Sub-adult leatherbacks had more variable headings and did not disperse as far south, with most turtles remaining north of 15°N (Fig. 2). Two sub-adults (Turtles K & S) were tracked near land (the Bahamas and the Lesser Antilles) during a portion of their migrations, but most sub-adults occupied offshore tropical and subtropical habitat during winter and early spring. The four largest adult males (Turtles I, L, N, T) and two adult females (Turtles H & M) traveled to areas off nesting beaches in the Lesser Antilles and Central America where they remained until tag transmissions ceased. Two smaller adult males and one adult female did not travel to known breeding areas; one male (Turtle F) remained in the Gulf Stream into early January and the other two turtles (Turtles G & P) overwintered in a tropical region near the convergence of the North Equatorial Current and North Equatorial Counter-Current.

## Discussion

We deployed GPS-linked and conventional ARGOS STDRs to simultaneously collect data on movements and dive behavior of adult and sub-adult leatherbacks in the North Atlantic. This study is one of the first to obtain highly accurate GPS locations from leatherback turtles, allowing us to identify high use habitat, movement patterns and environmental associations with less observation error. We also used novel design (e.g. “ridge-mount tag”) and direct attachment techniques to deploy low profile, hydrodynamic tags. In an analysis of transmitter drag and tag attachment procedures, Jones et al. [75] found that Wildlife Computers ridge-mount tags directly attached to the leatherback medial ridge resulted in the least amount of drag (0.6–1.8% increase in drag coefficient) whereas telemetry studies employing backpack harnesses resulted in the greatest amount of drag (92–112% increase in drag co-efficient). Field studies comparing the behavior of harnessed leatherbacks and leatherbacks with directly attached tags found significant differences in leatherback swim speed [51], [76] and dive duration [76]. We believe our tag model and tag attachment methods minimized impacts to the turtles' natural behavior, resulting in a more accurate portrayal of leatherback behavior than studies using harness attachments.

### Dispersal patterns and seasonal habitat use

Leatherbacks tagged off Massachusetts showed a strong affinity to the Northeast US continental shelf before dispersing widely throughout the northwest Atlantic. One individual tracked for >1 year exhibited site fidelity to the US shelf, returning in late spring and remaining through late summer. Surprisingly, only one Massachusetts-tagged leatherback moved onto the eastern Canada shelf, an important and well-documented leatherback foraging ground [16], [32], [77]–[78]. In contrast, leatherbacks tagged off eastern Canada spent extended periods in both Canadian and Northeast US shelf waters within a single foraging season [16]. Over half of the turtles tagged off Massachusetts were classified as sub-adults (55%) whereas sub-adults were the minority in the Canada tagging study (16%, n = 38). The sub-adults in our study may have been at the northern part of their foraging range. Migration distance depends partly on an animal's body size and their capacity to store energy (e.g., adipose tissue) [79]. Larger animals are able to swim longer distances at lower energetic cost [80], as has been predicted for cod [81] and bluefin tuna [82]. Smaller body size and lower lipid stores may limit the migratory range of some sub-adult leatherbacks. Larger leatherbacks also have an enhanced capacity for retaining metabolically produced heat and heat they absorb from their environment as a result of a small surface area to volume ratio and greater peripheral insulation [83]–[84]. Their superior thermoregulatory abilities would allow larger adult leatherbacks to more effectively exploit the cooler waters of the Canadian shelf than sub-adults. Alternatively, resources may have been sufficient on the Northeast US shelf during the years of our study, precluding a longer migration to more northerly foraging areas. Adult female leatherbacks tagged on nesting beaches in the northwest Atlantic showed different habitat utilization patterns than those tagged in temperate foraging grounds: high-use habitat tended to occur close to the nesting beaches where turtles were tagged or turtles were more widely distributed in oceanic and neritic regions throughout the North Atlantic [85]. Although there is inherent bias in determining habitat utilization from tracking data from one deployment location, bias is reduced with increasing deployment time, and alternative methods may be used to address this in the future [86]–[87].

There was a strong seasonal component to habitat selection, with most leatherbacks remaining in temperate latitudes in the summer and early autumn and moving into subtropical and tropical habitat in the late autumn, winter and spring. This latitudinal shift is consistent with previous studies of leatherbacks tracked from foraging grounds in the North Atlantic [24], [32] and similar to seasonal migration patterns of other sea turtle species [88]–[89] and large pelagic species such as ocean sunfish *Mola mola* [90], basking sharks *Cetorhinus maximus* [91], bluefin tuna *Thunnus thynnus* [92]–[93], and swordfish *Xiphias gladius* [94]. Latitudinal shifts in habitat-use likely reflect seasonal changes in temperature, productivity and prey availability at high latitudes, and, for some species, the necessity to spawn or nest in tropical regions. At temperate latitudes, gelatinous zooplankton populations are highest during summer months following the spring phytoplankton bloom, and large scyphozoan species may be more abundant in these cooler, temperate waters due to enhanced trophic transfer efficiencies [26]. These factors likely drive leatherbacks' seasonal exploitation of temperate coastal habitat, while their thermoregulatory and breeding constraints compel them to return to subtropical and tropical habitat from late autumn to spring [26].

During the over-wintering period, sub-adults, small adult males, and a single inter-nesting-year female primarily remained in oceanic habitat, while large adult males and two reproductive females moved into coastal breeding areas. Little is known about the demographics of male leatherbacks, but there may be a size constraint whereby smaller males are unable to compete for females, and are effectively displaced from breeding areas by larger, more dominant individuals [79]. Smaller males may direct energy toward growth rather than reproduction, and select over-wintering habitat to maximize limited foraging opportunities in the tropics. Adult females in an inter-nesting year are likely to avoid breeding areas where they would be subject to aggressive mating attempts by males [95]; by overwintering offshore, they can save energy and accumulate fat stores for return migration and future reproductive effort. Sub-adults also largely avoided breeding areas and most did not travel as far south as adults. The highly oriented paths taken by adult leatherbacks suggests movement toward a goal (e.g., specific breeding and/or foraging areas), while the more variable headings taken by sub-adults may indicate an opportunistic overwintering strategy, or lack of experience locating consistent resource patches in oceanic habitat.

### Regional movements, dive behavior, and habitat characteristics

#### Temperate neritic habitat

Our density utilization maps demonstrate that the Northeast US shelf, particularly southern New England, provides important seasonal habitat for leatherback turtles tagged of Massachusetts. The Northeast US shelf is one of the most well-studied and productive large marine ecosystems in the world [1], [96]. The region is characterized by a nutrient-limited, spring production peak; increasing irradiance and stratification in spring lead to a phytoplankton bloom, with peak surface chl *a* biomass in April [1]. The spring bloom in this productive coastal ecosystem is dominated by large cell phytoplankton (diatoms), likely leading to increased trophic transfer efficiencies (reviewed by Saba [26]). Food resources resulting from the predictable bloom feed juvenile gelatinous zooplankton that will be become fully mature by summer when leatherbacks are present [26], [97]. In addition to the high productivity of the system, the Northeast US shelf provides the hard bottom substrate needed by the benthic polyp stage of most schyphozoans [26], [97], the favored prey of leatherbacks in coastal waters of the Northeast US [57] and eastern Canada [78].

Previous studies have identified the Northwest Atlantic Shelves as important foraging habitat for leatherbacks [16], [26], [33], [57], [77], [98], and we found their behavior (slow, sinuous swimming) in this region to be consistent with area-restricted search. Leatherback locations in the Northwest Atlantic Shelves coincided with higher surface chl *a* and stronger SST gradients (with the exception of the Gulf Stream) relative to other regions, and most dives were in the euphotic zone within the average seasonal mixed layer depth on the Northeast US shelf (10–20 m) [1]. Highly productive water masses and frontal zones influence the spatial distribution and movements of some top predators, aggregating them in relatively small areas or “hotspots” on the shelf, shelf break, slope, offshore and at depths where prey is concentrated [36], [38], [99]–[100]. Other sea turtle species associate with enhanced frontal activity [101]–[102], though some cheloniids may face thermal constraints, limiting their access to cooler temperate frontal zones [102]. Weak winds and strong stratification are typical summer conditions on the Northeast US shelf that could lead to increased retention of gelatinous organisms [26]. Leatherback movements coincided spatially and temporally with the persistent Shelf-Slope Front and tidal-mixing fronts north of Nantucket Shoals, in the Gulf of Maine, and around Georges Bank [103]. The tidal-mixing fronts occur during peak leatherback presence in summer and early autumn, and may play an important role in consolidating seasonally abundant patches of the leatherback's gelatinous prey. Our model results showed that leatherbacks increased path sinuosity at shallower depths and warmer surface temperatures within the Northwest Atlantic Shelves. Decreasing water depth has been linked to increases in epipelagic gelatinous zooplankton biomass on a global scale, with greatest biomass found in shallow locations (<10 m average depth) [59]. Shallow shelf habitat such as shoals, banks, and ledges may be important for leatherback prey searching and (or) foraging in this region, and is consistent with our field observations of leatherbacks feeding in shoal habitat off Massachusetts [57] (Fig. 1).

The average mixed layer depth on the Northeast US shelf is also shallowest (10–20 m) during the summer and early autumn [1], potentially aggregating gelatinous prey at or above the pycnocline [104]-[105]. This would reduce ascent and descent times for foraging leatherbacks, and minimize time spent in cool waters below seasonal thermoclines. The percentage of time that leatherbacks spent at the surface in the Northwest Atlantic Shelves (27%) was much lower than that observed by James et al. [106] for turtles tagged off eastern Canada (mean 43% night and 50% day, n = 12 (0–2 m) and n = 3 (0–3 m)). The Canada-tagged turtles used habitat off the Northeast US as well as eastern Canada, so the disparity in percent surface times is somewhat surprising, but the colder water temperatures experienced by leatherbacks off eastern Canada may contribute to increased surface times in that region. James et al. [32], [106] described regular observations of leatherbacks basking at the surface off Nova Scotia, a behavior that we rarely observed off Massachusetts. It is possible that leatherbacks off eastern Canada spend greater time at the surface for thermoregulation [32] but this behavior is less important in the comparatively warm waters of the Northeast US shelf.

#### Subtropical oceanic habitat

We observed marked behavioral changes as leatherbacks left continental shelf habitat and began their southward migrations through subtropical oceanic habitat. As turtles moved through the Gulf Stream and Subtropical Atlantic, they showed rapid, directed travel and began spending more time at depths >50 m. Most turtles spent minimal time in these ecoregions, suggesting that these are less important feeding areas for Massachusetts-tagged leatherbacks and are primarily used for transiting between temperate (i.e., foraging) and tropical (i.e., breeding) habitat. However, two individuals did make more extensive use of the Gulf Stream region during summer, fall and early winter. The Gulf Stream's strong horizontal SST gradient, particularly in fall and winter, is evident in the strong SST fronts encountered by leatherbacks there. Leatherback movements in the Gulf Stream became slightly more sinuous at lower SST, possibly associated with upwelling along the Gulf Stream front, but this relationship was weak in our model. The Gulf Stream has been previously identified as probable foraging habitat for leatherback turtles [20], [25], [49], and turtles exploiting this area may take advantage of the enhanced productivity of energetic, mesoscale eddies characteristic of this region [1].

Leatherback movement patterns and dive behavior in the Subtropical Atlantic were consistent with other studies of this species in the North Atlantic [22], [25], [32], [49], [106]. Observed changes in dive behavior in the Subtropical Atlantic may be partially explained by cycles of seasonal stratification and depth of the mixed layer and (or) deep chlorophyll maximum. In the Subtropical Atlantic, the mixed layer deepens in the fall and winter when leatherbacks are present, and average mixed layer depth is over 50 m [1]. If leatherbacks target the pycnocline to search for prey, they would increase their diving activity to depth strata >50 m, with longer durations associated with increasing ascent and descent times. Leatherbacks in Subtropical Atlantic may also be exploiting the intense trophic activity associated with the deep chlorophyll maximum, which occurs at about 100 m [1]. Consumption at the deep chlorophyll maximum follows a strong diel cycle [1], and leatherbacks may be capitalizing on enhanced nighttime prey availability by making deeper, longer dives [106]. However, the rapid transit rate and limited time spent in the Subtropical Atlantic suggests that this region is not as important to over-wintering leatherbacks as tropical regions. Reproductive adults would have additional incentive to move quickly through the Subtropical Atlantic to reach breeding and nesting areas in the tropics.

The percentage of time that leatherbacks spent at the surface in the Gulf Stream and the Subtropical Atlantic was similar to the percent surface times recorded by James et al. [106] in the morning (06:00–12:00 GMT; mean 29%) and evening (18:00–0:00 GMT; mean 29%) periods of the southern migration, but much lower than their percent surface time during the day (12:00–18:00 GMT; mean 77%). Considering only the day period in our data set (12:00–18:00 GMT), the average percent surface time is still much lower (44%). Turtles tagged off eastern Canada and Massachusetts had similar dispersal and migratory patterns in the subtropical gyre, so the observed difference in surface times may be due to other factors such as different demographics (e.g., sex and body size) of our turtle sample or tagging technique (harness vs. direct attachment). Comparisons of these techniques showed that leatherbacks had lower travel rates [51], [76] and shorter dive durations [76] when wearing a harness. While surface time was not directly addressed in these comparisons, it's possible that leatherbacks wearing harnesses increase their surface time to recover from the energetic costs of increased drag caused by the harness (91–112% increase in drag coefficient) [75].

#### Tropical oceanic and neritic habitats

Leatherbacks overwintered in tropical ecoregions, with reproductively active adults primarily occupying the Guianas Coastal and Caribbean regions while non-reproductively active adults and sub-adults mainly used oceanic habitat in the Tropical Atlantic. Turtles slowed down in the tropics compared to the subtropical gyre but travel was still directed compared to the sinuous movements we observed in the Northwest Atlantic Shelves, suggesting a mix of behaviors that may include foraging, transiting and breeding. The Guianas Coastal and Caribbean regions encompass important breeding and nesting habitat for leatherbacks [85]. Two adult females in our study nested in the Guianas Coastal region (Trinidad and Costa Rica/Panama) [57], and two adult males remained in coastal waters off Trinidad. In the primarily shallow, shelf Guianas Coastal region, leatherback movements became more sinuous in response to decreasing water depth, probably linked to breeding activity [16], [22], [107] rather than feeding [108], although some leatherbacks do forage during the nesting season [109]–[111].

The average mixed layer depth varies throughout the tropics, with deepest depths occurring in winter when leatherbacks are present [1]. Leatherbacks occupied the western side of the Tropical Atlantic where average winter mixed layer depths are 70–80 m [1]. This could explain the deeper, longer dives that leatherbacks made there if prey accumulates near the pycnocline and (or) nutricline [112]–[113]. In the Tropical Atlantic, the night-time depths of diel migrants is consistently within the upper 50–75 m [1], and leatherbacks may target prey aggregated in this layer [106], though nocturnal foraging may be light-limited based on studies of leatherback ocular morphology [114] and feeding behavior [110].

The convergence of the North Equatorial Current, North Equatorial Counter-Current and the North Brazil Current appears to play an important role for overwintering leatherbacks in the southern part of their range. From June to January, the upper North Brazil Current joins the meandering North Equatorial Counter-Current at a retroflection zone near 5–10°N, where large, anti-cyclonic eddies are formed [115]. The eddies, known as North Brazil Current rings, have loop diameters of hundreds of kilometers, with a lifespan on the order of months, and they propagate northwest along the Brazil coast towards the Lesser Antilles [115]–[116]. North Brazil Current rings, and the convergence of the westward-flowing North Equatorial Current and eastward-flowing North Equatorial Counter-Current at 10–12°N, are associated with enhanced wintertime productivity [1]. Three leatherbacks resided in this region during winter months, associating with meanders from the North Equatorial Current and Counter-Current convergence and mesoscale eddies. Fossette et al. [25] inferred high foraging success year-round for leatherbacks at the southern boundary of the Tropical Atlantic, while a single tagged leatherback resided in this area for several months [117]. Since overall production in open tropical oceans is low compared to temperate and boreal latitudes [1], leatherbacks may rely on enhanced productivity there to maximize foraging opportunities during overwintering periods in the tropics.

Both adult and sub-adult leatherbacks in our study adjusted their movements and dive behavior in response to regional differences in environmental features. Leatherbacks increased their path sinuosity with decreasing water depth in temperate and tropical shelf habitats. This relationship is consistent with increases in gelatinous zooplankton biomass with decreasing water depth [59], and bathymetry may be a key feature in identifying leatherback foraging habitat in neritic regions. Coastal ecosystems are under intense pressure worldwide, with some of the highest predicted cumulative impact in the North American eastern seaboard and the eastern Caribbean [118]. Parts of these regions constituted high-use habitat for leatherbacks in our study, putting turtles at heightened risk from both land- and ocean-based human activity.
